# The role for the metagenome in the pathogenesis of COVID-19

**DOI:** 10.1016/j.ebiom.2020.103019

**Published:** 2020-10-07

**Authors:** Robert P Friedland, Bodduluri Haribabu

**Affiliations:** a Department of Neurology, University of Louisville School of Medicine, Louisville, KY 40202, United States; b Department of Microbiology and Immunology, University of Louisville School of Medicine, Louisville, KY 40202, United States

The novel corona virus SARS-CoV-2 infection and the COVID-19 pandemic has become an unrelenting worldwide catastrophe of public health and an economic emergency. A key question concerning COVID-19 is why most infected persons do not develop severe disease, while others become critically ill [1]. We know that this dichotomy is related to age, gender, immunosuppression and comorbidities [1]. But many persons who are young succumb to the virus, and we need to know why. In a relatively short time, several major advances in the field led to the decoding of the viral genome, and modes of transmission, as well as mechanisms of immune response to the infection that mostly succeeds in clearance [2]. However, a significant percent of cases develop runaway inflammation that fails to clear the infection and result in sepsis like multi-organ failure and death [3]. Unlike SARS and MERS infections that result in higher morbidity, COVID-19 manifests in a highly variable response based on the pathophysiological status of the host. *We propose that the metagenome directly contributes to this variable response* (Fig. 1).

**Fig. 1:**
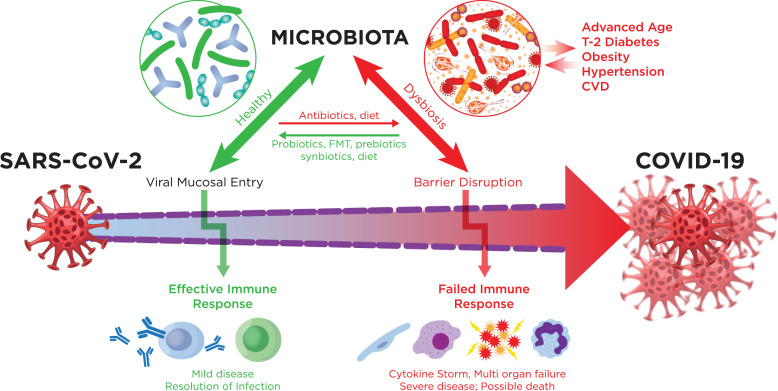
The potential role of the microbiota in COVID-19. Infection with SARS-CoV2 can cause excessive inflammation with enhanced production of cytokines and interferons leading to severe disease with poor outcomes. The virus may also cause epithelial barrier dysfunction enhancing damaging inflammatory responses. Dysbiosis in the gut, nose, oropharynx and lungs may initiate and worsen these pathogenic processes. The well-known comorbidities of COVID-19 are all associated with dysbiosis. On the other hand, healthy microbiota may inhibit the development of excessive inflammation and enhance an effective immune response, leading to better outcomes. Preventive and treatment strategies can be developed to enhance the health of our microbial populations, improving results.

Age and metabolic disorders such as obesity and type 2 diabetes are major risk factors for COVID-19 severity [4]. A common factor associated with aging and other COVID-19 risk factors is the dysbiosis of gut microbiota and resulting low grade inflammation with loss of epithelial barrier function [5]. An early study by Li et al. in Wuhan, reported a mean interval of 9.1–12.5 days between the onset of illness and hospitalization [6]. This delay in the progression to serious disease suggests that the pathogenesis of COVID-19 involves host specific factors that provide a unique window of opportunity for intervention. One possibility is that epithelial destruction caused by the binding of the virus to ACE2 receptors on gut enterocytes adds to the barrier dysfunction associated with comorbidities such as aging, obesity and heart disease. The resulting activation of the immune system due to pathogenic gut microbes results in a nonproductive innate immune response, as well as suppression of the adaptive immune response. There are several ways in which the microbiota influences this pathogenic immune process[7], [8], [9], [10]. Germ free animals have defective immune systems and the gut microbiota influences pathogen dissemination, inflammation, organ damage and mortality in murine pneumonia [9]. The microbiota also alters the efficacy of vaccines. Furthermore, a high-fiber diet enhances the growth of bacteria that make FOXP3 inducing short-chain fatty acids, which epigenetically enhance production of regulatory lymphocytes (Treg cells) which are effectively anti-inflammatory [8]. Short chain fatty acid production is also linked to the integrity of the intestinal barrier. Changes in diet with aging may well influence short chain fatty acid production, affecting immune homeostasis, barrier function and severity of COVID-19. Also, commensal microbiota modulates interferon production in the lung and it has been demonstrated that the microbiota influence TLR- augmented immune responses in a mouse model of the cytokine storm [10]. Recent studies showed that nearly 50% of the general population have a T-cell response to SARS-CoV-2 due to cross-reactivity to common cold viruses, thus explaining large numbers of asymptomatic carriers of the virus[11,12]. These observations suggest that lasting cross reactivity to common cold viruses might explain, at least in part, the high degree of variation in the severity of COVID-19. However, in cases of severe disease of COVID-19, it is the innate response and not the unregulated adaptive immune response via T cells that results in morbidity and death.

Although SARS-CoV-2 has been shown to infect the GI tract and may be excreted and transmitted through stool, the oral, nasopharyngeal and lung microbiomes may also play a vital role in accelerating COVID-19 pathogenesis. In this regard, it is interesting to note that oral pathogens were directly shown to influence colitis in mouse models, suggesting remote control of inflammation [13]. It is well established that the loss of epithelial barrier function at any mucosal site may initiate systemic dissemination, as well as remote organ destruction. Furthermore, many critically ill COVID19 patients are receiving antibiotics and have drastically altered dietary input, which will both have critical influences on microbial populations in the gut

It is important to appreciate the potential influence of the microbiota on COVID19 infections because there are many ways in which the microbial populations we possess can be altered, involving diet, antibiotics, prebiotics, probiotics, synbiotics, supplements and fecal microbiota transplants. The influence of the microbiota on immune processes in COVID19 infection may be assessed with metagenomic analysis of nasal, oral and intestinal communities, as well as metabolomics. It may be that uninfected subjects at risk, as well as infected persons, can take preventive measures designed to alter the microbiome to lower their risk of developing severe complications of COVID19 pneumonia, in addition to other viral disorders. The wide range of coronavirus investigations underway should consider the myriad potential influences of the microbiota and microbial metabolites on the illness.

## References

[1] Yang X., Yu Y., Xu J., Shu H., Xia J., Liu H., Wu Y., Zhang L., Yu Z., Fang M., Yu T., Wang Y., Pan S., Zou X., Yuan S., Shang Y (2020). Clinical course and outcomes of critically ill patients with SARS-CoV-2 pneumonia in Wuhan, China: a single-centered, retrospective, observational study. Lancet Respir Med.

[2] García L.F (2020). Immune response, inflammation, and the clinical spectrum of COVID-19. Front Immunol.

[3] McKechnie JLB, C.A. (2020). The innate immune system: fighting on the frontlines or fanning the flames of COVID-19?. Cell Host and Microbe.

[4] Petrilli C.M., Jones S.A., Yang J., Rajagopalan H., O'Donnell L., Chernyak Y., Tobin K.A., Cerfolio R.J., Francois F., Horwitz L.I (2020). Factors associated with hospital admission and critical illness among 5279 people with coronavirus disease 2019 in New York City: prospective cohort study. BMJ.

[5] Tilg H., Zmora N., Adolph T.E., Elinav E (2020). The intestinal microbiota fuelling metabolic inflammation. Nat Rev Immunol.

[6] Li Q., Guan X., Wu P., Wang X., Zhou L., Tong Y., Ren R., Leung K.S.M., Lau E.H.Y., Wong J.Y., Xing X., Xiang N., Wu Y., Li C., Chen Q., Li D., Liu T., Zhao J., Liu M., Tu W., Chen C., Jin L., Yang R., Wang Q., Zhou S., Wang R., Liu H., Luo Y., Liu Y., Shao G., Li H., Tao Z., Yang Y., Deng Z., Liu B., Ma Z., Zhang Y., Shi G., Lam T.T.Y., Wu J.T., Gao G.F., Cowling B.J., Yang B., Leung G.M., Feng Z (2020). Early transmission dynamics in Wuhan, China, of novel coronavirus-infected pneumonia. N Engl J Med.

[7] Bradley K.C., Finsterbusch K., Schnepf D., Crotta S., Llorian M., Davidson S., Fuchs S.Y., Staeheli P., Wack A (2019). Microbiota-driven tonic interferon signals in lung stromal cells protect from influenza virus infection. Cell Rep.

[8] Furusawa Y., Obata Y., Fukuda S., Endo T.A., Nakato G., Takahashi D., Nakanishi Y., Uetake C., Kato K., Kato T., Takahashi M., Fukuda N.N., Murakami S., Miyauchi E., Hino S., Atarashi K., Onawa S., Fujimura Y., Lockett T., Clarke J.M., Topping D.L., Tomita M., Hori S., Ohara O., Morita T., Koseki H., Kikuchi J., Honda K., Hase K., Ohno H (2013). Commensal microbe-derived butyrate induces the differentiation of colonic regulatory T cells. Nature.

[9] Schuijt T.J., Lankelma J.M., Scicluna B.P., de Sousa e Melo F., Roelofs J.J., de Boer J.D., Hoogendijk A.J., de Beer R., de Vos A., Belzer C., de Vos W.M., van der Poll T., Wiersinga W.J (2016). The gut microbiota plays a protective role in the host defence against pneumococcal pneumonia. Gut.

[10] Weaver L.K., Minichino D., Biswas C., Chu N., Lee J.J., Bittinger K., Albeituni S., Nichols K.E., Behrens E.M (2019). Microbiota-dependent signals are required to sustain TLR-mediated immune responses. JCI Insight.

[11] Grifoni A., Weiskopf D., Ramirez S.I., Mateus J., Dan J.M., Moderbacher C.R., Rawlings S.A., Sutherland A., Premkumar L., Jadi R.S., Marrama D., de Silva A.M., Frazier A., Carlin A.F., Greenbaum J.A., Peters B., Krammer F., Smith D.M., Crotty S., Sette A (2020). Targets of T cell responses to SARS-CoV-2 coronavirus in humans with COVID-19 disease and unexposed individuals. Cell.

[12] Mateus J., Grifoni A., Tarke A., Sidney J., Ramirez S.I., Dan J.M., Burger Z.C., Rawlings S.A., Smith D.M., Phillips E., Mallal S., Lammers M., Rubiro P., Quiambao L., Sutherland A., Yu E.D., da Silva Antunes R., Greenbaum J., Frazier A., Markmann A.J., Premkumar L., de Silva A., Peters B., Crotty S., Sette A., Weiskopf D (2020). Selective and cross-reactive SARS-CoV-2 T cell epitopes in unexposed humans. Science.

[13] Kitamoto S., Nagao-Kitamoto H., Jiao Y., Gillilland M.G., Hayashi A., Imai J., Sugihara K., Miyoshi M., Brazil J.C., Kuffa P., Hill B.D., Rizvi S.M., Wen F., Bishu S., Inohara N., Eaton K.A., Nusrat A., Lei Y.L., Giannobile W.V., Kamada N (2020). The intermucosal connection between the mouth and gut in commensal pathobiont-driven colitis. Cell.

